# Intestinal Sucrase as a Novel Target Contributing to the Regulation of Glycemia by Prebiotics

**DOI:** 10.1371/journal.pone.0160488

**Published:** 2016-08-17

**Authors:** Audrey M. Neyrinck, Barbara Pachikian, Bernard Taminiau, Georges Daube, Raphaël Frédérick, Patrice D. Cani, Laure B. Bindels, Nathalie M. Delzenne

**Affiliations:** 1 Metabolism and Nutrition Research Group, Louvain Drug Research Institute, Université catholique de Louvain, Brussels, Belgium; 2 Fundamental and Applied Research for Animal and Health (FARAH), Faculty of Veterinary Medicine, University of Liège, Sart Tilman, Liège, Belgium; 3 Medicinal Chemistry Research Group, Louvain Drug Research Institute, Université catholique de Louvain, Brussels, Belgium; 4 Walloon Excellence in Life sciences and BIOtechnology (WELBIO), Louvain Drug Research Institute, UCL, B-1200 Brussels, Belgium; Western University of Health Sciences, UNITED STATES

## Abstract

Inulin-type fructans (ITF) are known for their capacity to modulate gut microbiota, energy metabolism and to improve glycemia in several animal models of obesity, and in humans. The potential contribution of ITF as modulators of sugar digestion by host enzymes has not been evaluated yet. A sucrose challenge has been performed on naive mice fed a standard diet supplemented with or without native chicory inulin (Fibruline 5%) for 3 weeks. The area under the curve of glycemia as well as sucrase activity in the small intestine were lowered after inulin treatment. Pyrosequencing of the 16S rRNA gene confirmed important changes in gut microbiota (mostly in favor of *Blautia* genus) due to inulin extract supplementation. Interestingly, the suppressive effect of inulin extract on postprandial glycemia also occurred when inulin was directly added to the sucrose solution, suggesting that the effect on sucrose digestion did not require chronic inulin administration. *In vitro* tests confirmed a direct inhibition of sucrase enzyme by the inulin extract, thereby suggesting that native chicory inulin, in addition to its well-known prebiotic effect, is also able to decrease the digestibility of carbohydrates, a phenomenon that can contribute in the control of post prandial glycemia. We may not exclude that the sucrose escaping the digestion could also contribute to the changes in the gut microbiota after a chronic treatment with inulin.

## Introduction

Excessive post-prandial glucose excursions are a risk factor for developing diabetes associated with impaired glucose tolerance. Sugar intake is a matter of debate concerning its contribution to metabolic disorders, sucrose being the predominant sugar in processed foods and drinks. Sucrose is glucose linked to fructose and is hydrolysed by sucrase (sucrose alpha-glucosidase), an enzyme present in the intestinal mucosa as a single polypeptide chain [1,2]. After digestion, both fructose and glucose enter the bloodstream. The increase in the blood glucose concentration is followed by release of insulin which allows, in normal condition, a normalization of glycemia within 1–3 h.

Inulin-type fructans (ITF) have initially been recognized as prebiotics, because they are “nondigestible compounds that, through their metabolization by microorganisms in the gut, modulate composition and/or activity of the gut microbiota, thus conferring a beneficial physiological effect on the host” [3,4]. Even if relevant intervention studies with ITF in humans are rare, there are some data supporting the fact that prebiotics could be interesting in the management of diabetes. In the majority of studies, the chronic administration of prebiotics leads to an improvement of hepatic insulin resistance and fasting and/or post-oral glucose load glycemia [5–7]. Several mechanisms have been proposed to explain the beneficial effect of prebiotics in rodent models of obesity and diabetes. The modulation of the endocrine function occurring in prebiotic-fed animals is one of the pathways that contribute to the improvement of obesity and associated metabolic disorders [8–11]. Indeed, the ingestion of prebiotics has been shown to increase the number of entero-endocrine L cells in the proximal colon of rodents. The resulting increase in portal glucagon-like peptide 1 (GLP-1) leads to a regulation of food intake and glycemia since this peptide stimulates insulin secretion in a glucose-dependent manner [10,12,13]. Another factor that participates to the regulation of post-prandial glycemia is carbohydrate digestibility in the gut. However, the possible action of prebiotics on sugar digestibility via a modulation of disaccharidase activity has never been investigated until now. In the present study, we investigated the impact of native chicory inulin on sucrose digestibility after a long term feeding and after an acute intake. We aimed to explore the potential ability of inulin to inhibit sucrase activity in a microbiota-dependent and microbiota-independent ways. Therefore, we performed sucrose challenges in mice fed inulin for 3 weeks and in naïve mice to which inulin was co-administrated during the sucrose challenge. The gut microbiota compositon and the activity of sucrase from the jejunal mucosa were analyzed 4 weeks after inulin treatment. In addition, we used homogenates from the brush border membrane of the small intestine of naïve mice to investigate the inhibitory effect of inulin on sucrase activity and we compared this effect to the one of acarbose, a well known α-glucosidase inhibitor [2,14].

## Materials and Methods

### Mice and treatments

Eighteen male C57BL/6J mice (12-week-old) were purchased from Charles River Laboratories (France) and maintained in specific pathogen free environment. Animals were housed in groups of 3 mice per cage in a controlled environment (12-hour daylight cycle) with free access to food and water. After one week of acclimatization and 6h of fasting, a sucrose solution (3g/kg body weight) with or without inulin extract (5%, Fibruline®, Cosucra, Belgium) was orally administrated by gavage (150μl/10g body weight); the ratio sucrose:inulin (weight by weight) being 4:1. Blood glucose levels were determined using a glucose meter (Roche Diagnostics) on 3.5 μl of blood collected from the tip of the tail before and after sucrose solution administration. Sixty microliters of blood was also harvested 30 min before and 15 min after the sucrose load to assess plasma insulin concentrations. Then, mice receiving the sucrose solution without inulin extract were fed a standard diet (containing 10% sucrose, AIN93M, Research Diets) whereas the mice receiving the sucrose solution with inulin extract were fed the standard diet supplemented with 5% of the inulin extract (Fibruline®, Cosucra, Belgium). After 3 weeks of dietary treatment, another sucrose challenge was performed as described above without any inulin extract addition in the sucrose solution. After 4 weeks of dietary treatment, 6h-fasted mice were anaesthetized with isoflurane gas (Abbot, Ottignies, Belgium) and necropsied after cervical dislocation. Intestine segment from the jejunum was immediately removed, and the mucosa was scraped off, frozen in liquid nitrogen and stored at −80°C until the assay of sucrase activity. Caecal content and tissue were weighted, frozen in liquid nitrogen and stored at -80°C.

### Ethics statement

All mouse experiments were approved by and performed in accordance with the guidelines of the local ethics committee for animal care of the Health Sector of the Université catholique de Louvain under the supervision of Prof. F. Lemaigre and Prof. JP Dehoux and under the specific agreement numbers 2010/UCL/MD/022. Housing conditions were specified by the Belgian Law of May 29, 2013 regarding the protection of laboratory animals (agreement number LA1230314). All surgeries were performed under anesthesia (isoflurane gas), and all efforts were made to minimize suffering

### Sucrase activity

Sucrase activity assays were performed using the jejunal mucosa according to the partially modified Dahlqvist's method [15,16]. Briefly, 20mg of scrapped mucosa was homogenized in NaCl 0,9%. Twenty μl of homogenates was kept at 37°C in the presence or not of 5μl of inulin extract solution (10%, 5% or 1%) or 5μl of acarbose solution (1%); 20 μl of substrate solution (0.056M of sucrose) was added. After 1h of incubation at 37°C, 25 μl Tris buffer (3M, pH7) was added to stop the reaction. The glucose produced after hydrolysis of sucrose was measured using a kit coupling enzymatic reaction and spectrophotometric detection of reaction end products (Diasys Diagnostic and Systems, Holzheim, Germany). The amount of protein in the intestinal samples was determined by Lowry’s method (Lowry et al., 1951). The sucrase activity was expressed as μmol of sucrose hydrolyzed into glucose/mg protein.min. The inhibition was evaluated *in vitro* with increasing concentrations of sucrose in the presence of different concentrations of inulin extract. The type of inhibition was determined by Lineweaver-Burk plot analysis of the data, which was calculated from the results according to Michaelis-Menten kinetics.

### Gut microbiota analyses

Genomic DNA was extracted from the caecal content using a QIAamp DNA Stool Mini Kit (Qiagen, Hilden, Germany) according to the manufacturer’s instructions, including a bead-beating step. 16S rDNA profiling, targeting V1-V3 hypervariable region and sequenced on Illumina MiSeq, and qPCR were performed as described previously [17] and in S1 Text.

### Statistical analysis

Results are presented as the mean ± SEM, or Whiskers plot with minimum and maximum, for bacteria. Statistical analysis was performed by unpaired Student t test using the GraphPad Prism software (version 5.00, GraphPad Software, San Diego, California, USA) except for gut microbiota analyses by pyrosequencing. Statistical differences of bacterial population relative abundance were highlighted with multiple unpaired t test coupled with a False Discovery Rate (Q value 1%) using GraphPad Prism software (version 6.00, GraphPad Software, San Diego, California, USA). AMOVA analysis was performed using MOTHUR V1.35. The results were considered statistically significant when p < 0.05.

## Results

### Long term supplementation with native chicory inulin decreases sucrase activity in the small intestine

**Fig 1:**
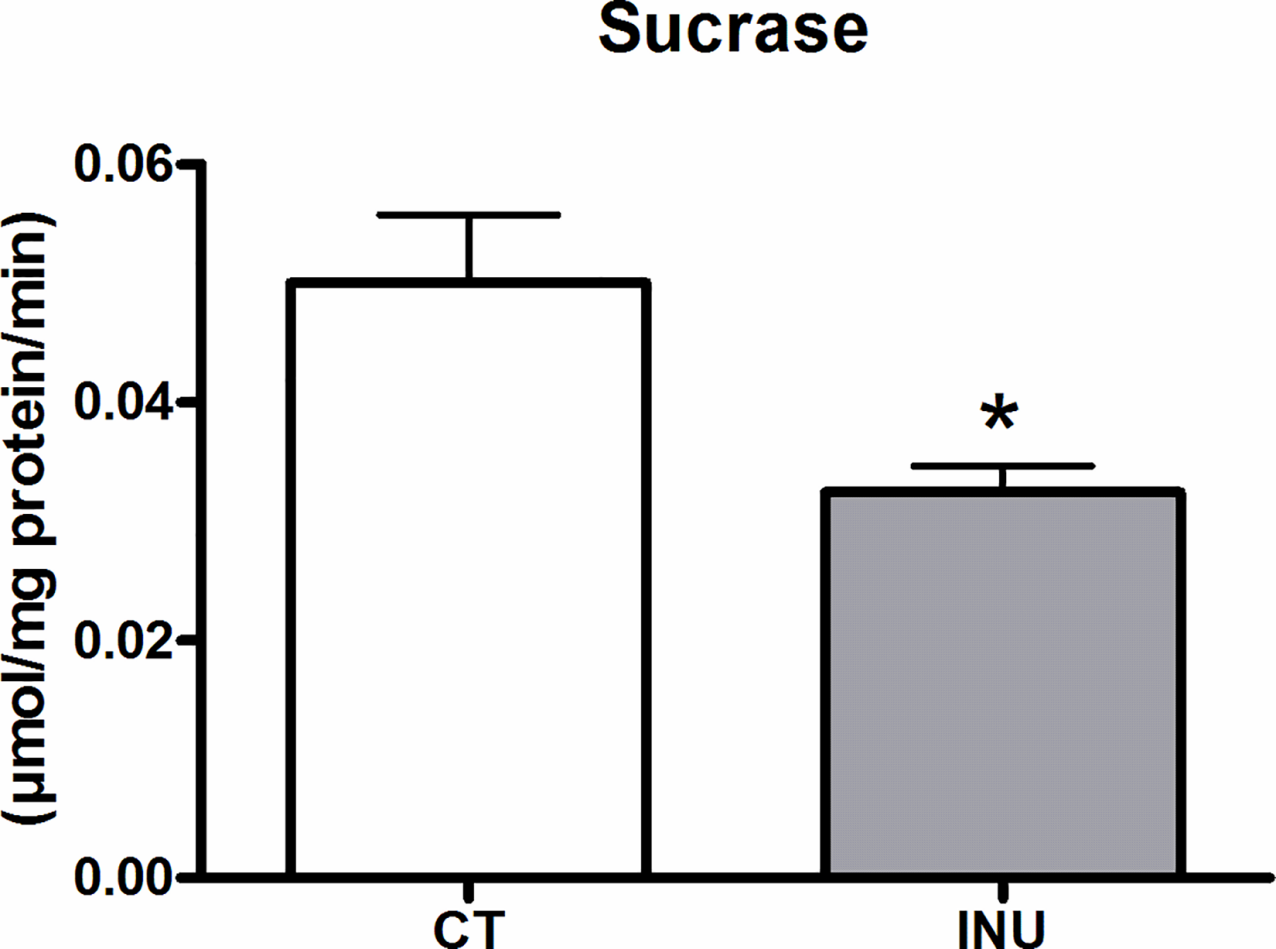
Sucrase activity in the small intestine. Mice fed a control diet (CT) or a diet supplemented with 5% inulin (INU) for 27 days. *p<0.05 (Student *t* test).

Sucrase activity was measured in the small intestinal mucosa 27 days after consumption of inulin extract and was significantly reduced upon inulin supplementation (Fig 1)

### Long term supplementation with native chicory inulin induces drastic changes in microbial populations

Inulin extract addition in the standard diet induced caecal fermentation as evidenced by higher caecal content and tissue weights after 27 days of dietary treatment (Table 1). This effect was not accompanied by changes in body weight gain, liver weight or fat mass.

**Table 1:** Body weight gain and organ weights.

	CT	INU
**Body weight gain (g)**	0.16 ± 0.46	0.25 ± 0.47
**Liver (g/100g body weight)**	3.70 ± 0.09	3.78 ± 0.05
**Caecal content (g)**	0.18 ± 0.01	0.26 ± 0.03*
**Caecal tissue (g/100g body weight)**	0.26 ± 0.01	0.36 ± 0.02*
**Adipose tissues (g/100g body weight):**		
- visceral	0.47 ± 0.04	0.54 ± 0.04
- epididymal	1.19 ± 0.10	1.18 ± 0.09
- subcutaneous	1.03 ± 0.09	1.02 ± 0.09

Mice were fed control diet (CT) or a diet supplemented with 5% inulin for 27 days.

*p<0.05 (Student *t* test).

**Fig 2:**
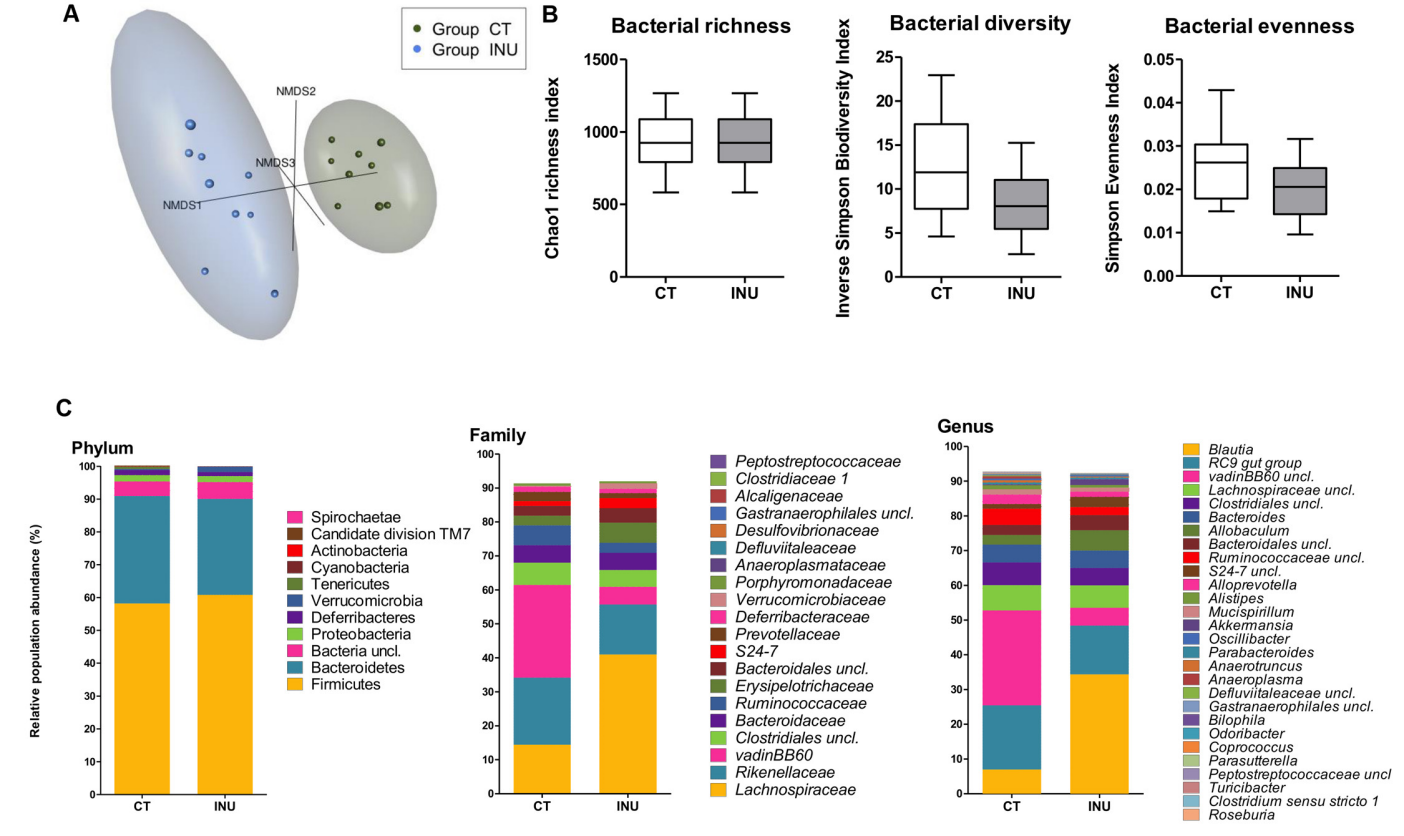
Spatial ordination, bacterial diversity and taxonomical distribution deduced by 16S profiling. Non metric dimensional scaling (three axis) with ellipse showing standard deviation (95% confidence) (A). Bacterial diversity (Inverse Simpson Biodiversity Index), bacterial richness (Chao1 Richness Index) and bacterial evenness (deduced from Simpson Index) (B). Mean Phylotype distribution (Phylum, family and genus levels) expressed as mean cumulated relative abundance (C). Mice were fed fed a control diet (CT, green) or a diet supplemented with 5% inulin (INU, blue) for 27 days.

The differences within the intestinal microbial population between the control group and the inulin extract-treated group were visualized by Non Metric Dimensional Scaling built upon a Bray-curtis distance matrix based on the species taxonomic level (Fig 2A). A distinct cluster was observed for mice receiving inulin extract supplementation as confirmed by AMOVA analysis of the distance matrix (p value <0.001). The inulin-treated mice exhibited a slight decrease in caecal microbial α-diversity compared to the control mice due to a lower evenness without significantly affecting the species richness; however none of these effects reached significance (Fig 2B). To determine which specific bacterial taxa were responsible for such a shift in the gut microbiota composition, we compared the relative abundance of bacterial taxa between groups (Fig 2C and S1 Table). We did not observe any shift between phyla. However, at the family level, our results showed an intra-phyla shift where the abundance of vadin BB60 group and *Ruminococcaceae* family decreased in favor of a higher abundance of *Lachnospiraceae* due to the inulin extract treatment. At a lower taxonomic level, the most prominent differences related to the *Blautia* genus, whose relative abundance reached 35% in inulin extract-fed mice. Unclassified bacteria from the *Ruminococcaceae* family and from vadin BB60 group decreased in inulin-treated mice. Of note, the abundance of *Akkermansia muciniphila* was not modified by the inulin supplementation under standard diet.

**Fig 3:**
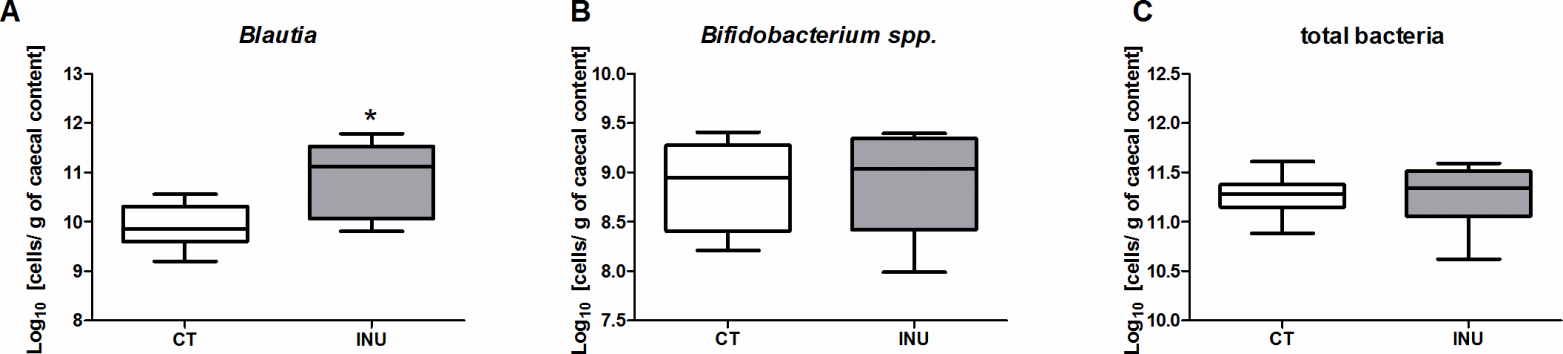
qPCR analysis of the caecal luminal microbiota. Levels of *Blautia* (A), *Bifidobacterium* spp. (B) and total bacteria (C) in the caecal content of mice fed a control diet (CT) or a diet supplemented with 5% inulin (INU) for 27 days. *p<0.05 (Student *t* test).

The effect on *Blautia* was confirmed by qPCR (Fig 3A). The inulin extract did not change the total number of bacteria in the caecal content and, surprisingly, it did not induce a bifidogenic effect (Fig 3B and 3C).

### Long term supplementation with native chicory inulin reduces postprandial glycemia without change of insulin levels upon sucrose challenge

**Fig 4:**
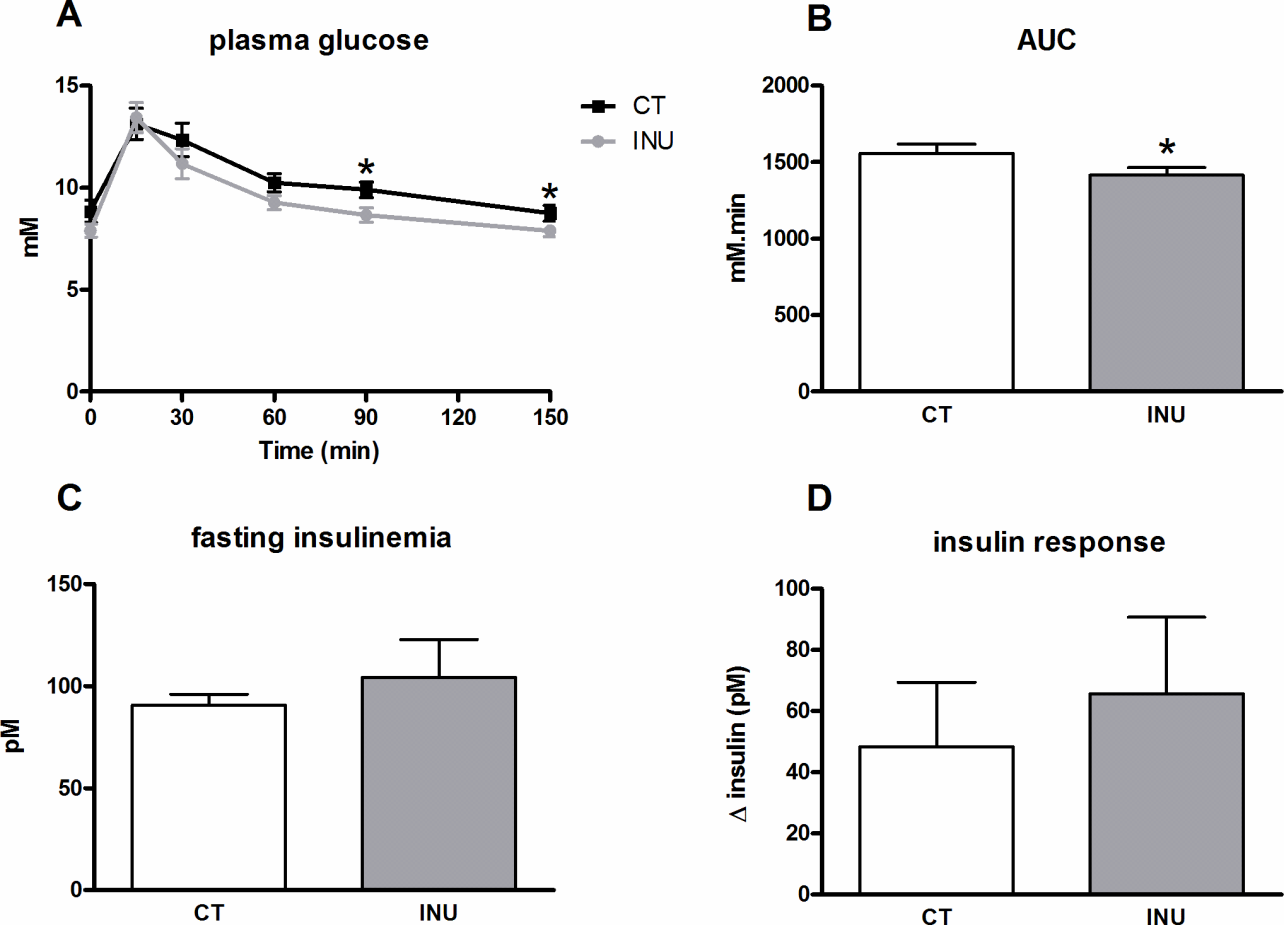
Effect of long term administration of inulin extract on postprandial blood glucose and serum insulin levels in sucrose-loaded mice. Mice were fed a control diet (CT) or a diet supplemented with 5% inulin (INU) for 3 weeks. Plasma glucose excursion after the oral sucrose load (A), area under the curve (AUC) of the glucose excursion (B), fasting insulinemia (C) and insulin response (Δ insulin concentrations 30 min before and 15 min after the sucrose load) (D). *p<0.05 (Student *t* test).

The effect of inulin supplementation on postprandial blood glucose level and serum insulin level was examined in sucrose-loaded mice after 3 weeks of dietary treatment (Fig 4). The peak of glycemia observed 15 min after the sucrose load was not modified by inulin extract (Fig 4A). In contrast, inulin-treated mice exhibited a lower postprandial glycemia 90 min and 120 min after the oral sucrose load, leading to a lower area under the curve for the glucose profile (Fig 4A and 4B). Fasting insulinemia and insulin response to the glucose load were not modified by the inulin supplementation (Fig 4C and 4D).

### Acute treatment with native chicory inulin reduces postprandial glycemia and insulinemia upon a sucrose challenge

**Fig 5:**
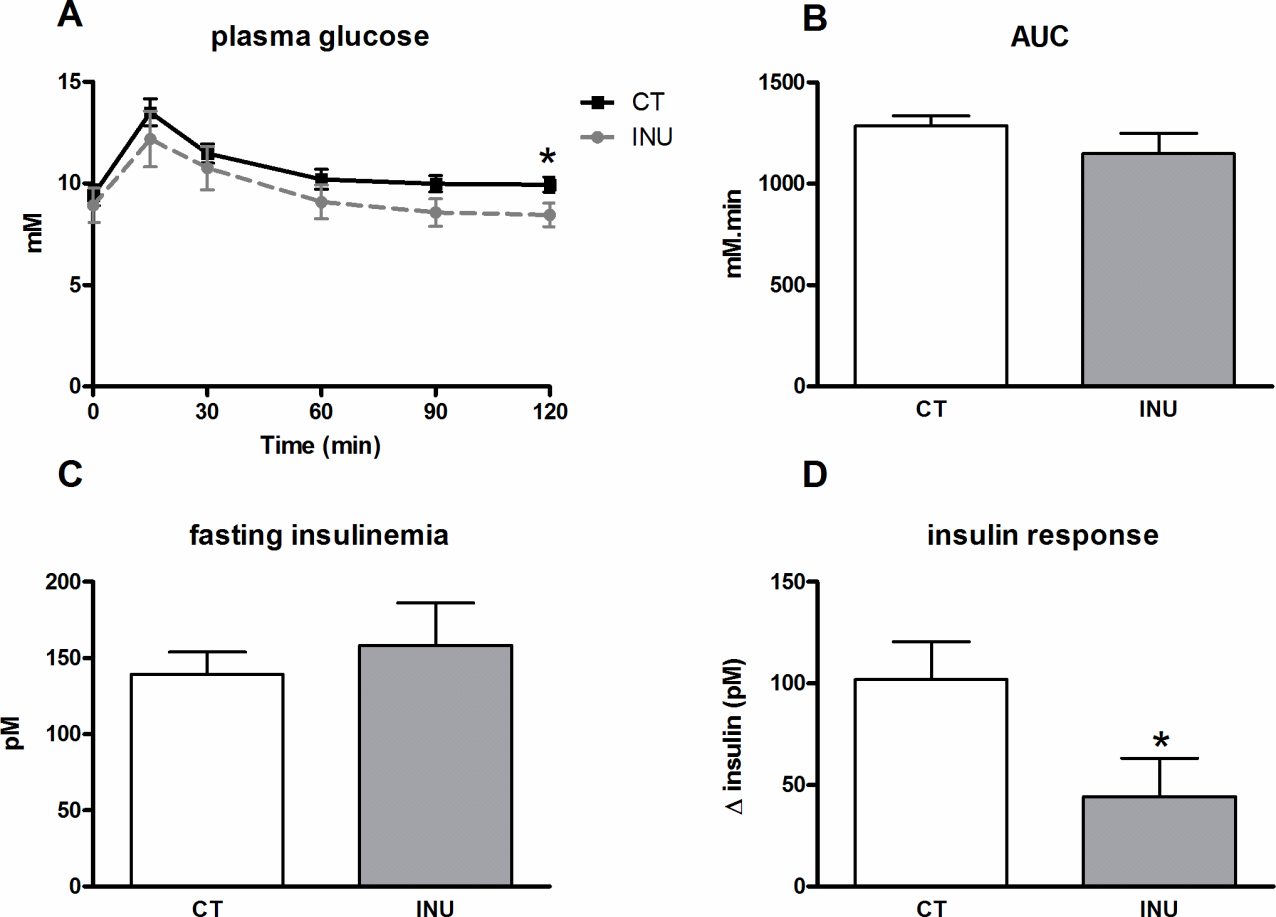
Effect of inulin extract addition (5%) in the sucrose solution on the postprandial blood glucose and serum insulin levels in sucrose-loaded mice fed a control diet. Plasma glucose excursion after the oral sucrose load (A), area under the curve (AUC) of the glucose excursion (B), fasting insulinemia (C) and insulin response (Δ insulin concentration 30 min before and 15 min after the oral sucrose load) (D). *p<0.05 (Student t test).

The direct effect of inulin extract on the postprandial blood glucose level and serum insulin level was examined after sucrose challenge in mice fed a standard diet (Fig 5). When inulin extract was orally administrated simultaneously with sucrose to the mice, the peak of glycemia observed 15 min after the sucrose load was slightly lower as compared to the glycemia observed after the sucrose solution administration alone (Fig 5A). Moreover, the presence of inulin extract in the sucrose solution significantly decreased postprandial glycemia 120 min after the oral challenge (Fig 5A). Although we observed a lower area under the curve calculated from glucose profiles obtained after sucrose administration in the presence of inulin extract, this result did not reach significance (Fig 5B). Interestingly, the insulin response of mice facing the sucrose load was significantly lower in the presence of inulin extract in the oral solution. (Fig 5D).

**Fig 6:**
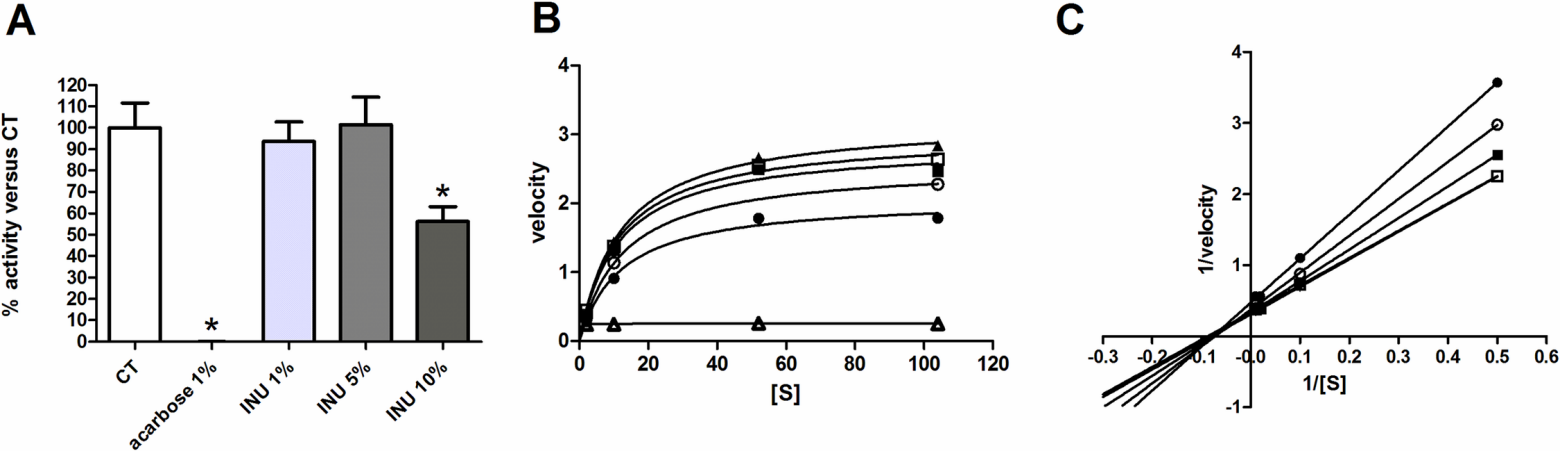
Sucrase activity *in vitro*. Inhibitory effect of inulin extract (INU) and acarbose on sucrase activity of small intestinal mucosa obtained from naïve mice (A). Michaelis-Menten (B) and Lineweaver-Burk (C) plots of the sucrase hydrolysis reaction with variable sucrose concentrations ([S] from 0 to104 mM) and at fixed concentration of inulin extract (0mM (▲), 3mM(□), 6mM(■), 11mM(○), 23mM(●), acarbose 5 mM(△)).

In order to evaluate the direct action of inulin extract on sucrase activity, we investigated its potential inhibitory effect *in vitro* using homogenates prepared from small intestinal mucosa of naïve mice (Fig 6). Acarbose was used as a known inhibitor of α-glucosidase. We observed an inhibitory effect of 44% for the higher concentration of inulin extract used (5 μl of a solution at 10%) (Fig 6A). To determine whether the inhibition of sucrase by inulin extract was competitive or non-competitive, Michaelis-Menten and Lineweaver-Burk plots were drawn. In the presence of inulin extract, the slope of the straight lines in a double reciprocal plot increased with increasing concentrations of inulin. The straight lines were intercepted at a single point in the second quadrant indicating mixed noncompetitive inhibition (Fig 6C).

## Discussion

Nutrition plays an important role in the management of energy metabolism, in particular glucose metabolism. Dietary fibers present in cereals, legumes or fruits are able to decrease the postprandial glucose response and to reduce glycemia and insulin responses in humans. Among these fibers, ITF has been shown to impact glucose levels (for review see [6]); The decrease in glycemia by ITF, when it replaces carbohydrates, has been recognized as a health effect by the European Food Safety Authority (EFSA) [18]. The antidiabetic effects of ITF are often linked to a higher secretion of gut peptides. Although the effects of several dietary fibers have been studied at the intestinal mucosal level [19–23], experimental work on ITF has mainly focused on their prebiotic effect due to fermentation in the large intestine. To our knowledge, no study has been conducted to evaluate its effect on disaccharidase activities of the small intestine. Our work shows that native inulin prepared from chicory root modifies sucrase activity measured in the scrapped mucosa of jejunum after 4 weeks of supplementation. The inhibitory effect on sucrase activity could participate to decrease the postprandial glycemia in sucrose-loaded mice fed a diet supplemented with inulin extract. Furthermore, the hypoglycemic effect of the inulin extract is not accompanied by changes in insulin levels, suggesting improved insulin sensitivity after chronic treatment. The insulin response observed is dependent on the glucose increase in the blood after the glucose load. The hypoglycemic effect of the inulin extract given together with the sucrose is accompanied by a logical lower insulin levels. The discrepancy between insulin level after acute versus chronic inulin treatment may be explained by changes in gut functions associated with a longer term treatment. Indeed, it was shown that prebiotic inulin-type fructans increased the insulin sensitivity after a chronic treatment in HF diet fed mice [10]. One of the potential explanations could be an increase in endocrine L cells in the jejunum and colon of rodents that promote the production and release of the active forms of GLP-1, which is able to increase insulin sensitivity [10,24]. Those effects are dependent on the modulation of the microbiota which takes place after a chronic treatment of at least 2 weeks and which was not considered 2h after an acute administration. In parallel, we observe a shift inside the Firmicutes phylum in favor of the genus *Blautia* from the *Lachnospiraceae* family, upon inulin extract supplementation as shown either by qPCR or pyrosequencing. Recent phylogenetic studies propose *Blautia* gen. nov., as a novel genus encompassing in particular *Clostridium coccoides* and several *Ruminococcus* species [25]. This genus, for which species have been isolated from animal and human feces, contains positive anaerobic bacteria having a fermentative type of catabolism whereas some other species use H_2_/CO_2_ as major energy sources. The major end products of glucose metabolism are acetate, ethanol, hydrogen, lactate and succinate. There are three major groups of H_2_-consuming microorganisms (hydrogenotrophs) in the human colon: methanogens, sulfate-reducing bacteria, and acetogens, and direct competition among them may occur for the common substrate H_2_ [26]. In our study, sulfate-reducing bacteria such as *desulfovibrionacaeae* (principally *Bilophila*) or methanogenic *Archaea* are less abundant (<0.2%) (whatever the group of mice, CT or INU) than the acetogenic *blautia* (>7%). The higher abundance of *Blautia* -reaching 35% due to inulin supplementation- could be beneficial in the context of obesity since it was shown that acetogens determine the biomass specific growth rate of the other hydrogenotrophs [27]. Interestingly, some of them -methanogenic *Archaea* and sulfate-reducing bacteria (*Bilophila*)- have been linked to fat feeding or altered metabolism, inflammation and weight gain [28–31]. Moreover, one study reported a *Blautia* species (*B*. *glucerasea*) as one of the main users of the ^13^C-labeled inulin added to the diet of rats [32]. In accordance with this finding, a cross-over trial with four-week treatments in which healthy humans consumed a daily dose of 60 g of whole-grain, reported an increase of the abundance of the genus *Blautia* in fecal samples in association with a decreased peak of postprandial glucose [33]. Surprisingly, inulin extract did not increase bifidobacteria in our study when it was added to the standard diet. Furthermore, the lack of reduction in body weight and fat mass could be due to the fact that the dose of 5% was used in the present study, rather than the dose of 10% usually used in most experimental data in mice of rats. However, we have already described a lack of significant effect of short chain inulin (different from the one used in this study) when added at 10% in the control diet [34,35]. It appears that the anti-obesity effect of inulin-type fructans associated with higher number of bifidobacteria is observed mostly upon HF feeding. Moreover the number (analyzed by qPCR, data not shown) and the abundance of *Akkermansia muciniphila* were not significantly modified by the inulin supplementation, as already observed under control diet in a previous study in which anti-obesity effect of prebiotic was evidenced only upon HF feeding [34]. Therefore, our pyrosequencing analyses, together with our previous analyses, reinforce the idea that microbial response to prebiotics can be individualized and that the microbial consequences of a prebiotic depend, among others, on the baseline composition of the microbiota [36,37].

The inhibitory effect of inulin extract on sucrase activity may be linked to change in gut microbiota since it was reported that its activity in the upper small intestine of germ-free animals was higher than the one in conventional or conventionalized animals [38,39]. However, we cannot exclude a direct effect of inulin extract on sucrase activity. Accordingly, we have demonstrated an inhibitory action for the inulin extract *in vitro* using small intestinal mucosa samples. Inhibition of sucrase activity by inulin extract was lower than acarbose, a known glucosidase competitive inhibitor, and its inhibitory mode for sucrose was mixed noncompetitive. To our knowledge, this is the first report showing an inhibition of sucrase activity by ITF. To determine if the suppressive effect of inulin on postprandial blood levels of glucose could result from an acute inhibition of sucrase activity, sucrose solution with or without inulin extract has been administered to mice. Increments in the blood levels of glucose and insulin are significantly suppressed after administration of the sucrose solution together with inulin extract to mice, in comparison to administration of the sucrose solution alone. These results suggest that inulin extract may have an inhibitory effect *in vivo* on sucrase activity and may through this mechanism, suppress the postprandial elevation of blood levels of glucose and insulin. The discovery of a new potential mechanism for inulin to regulate host metabolism independently of the gut microbiota reinforces the current hypothesis that prebiotic compounds can actually also exert some effects such as immunomodulation or anti-adherence by pathways that are independent of the gut microbiota [3,40,41].

In conclusion, our results suggest that native chicory inulin has an inhibitory effect on sucrase activity and may thereby exert a suppressive effect on the elevation of blood levels of glucose and insulin during sucrose ingestion in mice, independently of a chronic adaptation of the gut microbiota composition to inulin. Moreover, it would be interesting to evaluate if the carbohydrates that escape digestion and reach in the (caeco)colon could contribute to the bacterial changes in the caecum observed after chronic inulin administration. Our work suggests a promising role for native chicory inulin as a sugar replacer able to decrease the digestibility of carbohydrates and consequently post-prandial glycemia, and points out an interesting target to be measured in future clinical trials when looking for mechanisms linking inulin consumption with improved glucose metabolism.

## Supporting Information

S1 Table Abundance of bacteria taxa that are statistically impacted by the dietary treatment as determined by pyrosequencing of 16sRNA gene. (DOCX)

S1 Text Materials and Methods. (DOCX)
