# Supplementary material for: Intestinal Sucrase as a Novel Target Contributing to the Regulation of Glycemia by Prebiotics
Source: PLoS One. 2016 Aug 17;11(8):e0160488. doi: 10.1371/journal.pone.0160488 (PMC4988693; doi:10.1371/journal.pone.0160488)
Supplement: S1 Table — (DOCX) [file pone.0160488.s001.docx]

**S1 Table.** **Abundance of bacteria taxa that are statistically impacted by the dietary treatment as determined by pyrosequencing of 16sRNA gene.**

|  | Corrected p-value | Control  Mean (%) | Control  SD (%) | Inulin  Mean (%) | Inulin  SD (%) |
| --- | --- | --- | --- | --- | --- |
| **At the family level**  *vadinBB60 group* | 2.25E-04 | 27.289 | 8.012 | 5.137 | 4.297 |
| *Clostridiales_Family_XIII* | 6.92E-03 | 0.077 | 0.046 | 0.000 | 0.000 |
| *Ruminococcaceae* | 4.04E-02 | 5.811 | 1.952 | 3.012 | 1.064 |
| *Lachnospiraceae* | 4.20E-02 | 14.456 | 5.336 | 40.961 | 19.670 |
| **At the genus level**  *vadinBB60_unclassified* | 3.41E-04 | 27.289 | 8.012 | 5.137 | 4.297 |
| *Anaerovorax* | 3.08E-02 | 0.031 | 0.021 | 0.000 | 0.000 |
| *Family_XIII_unclassified* | 2.15E-02 | 0.046 | 0.031 | 0.000 | 0.000 |
| *Blautia* | 2.25E-02 | 6.985 | 2.565 | 34.398 | 19.040 |
| *Ruminococcaceae_unclassified* | 4.83E-02 | 4.706 | 1.762 | 2.329 | 0.667 |
| **At the species level (OTUs)**  *vadinBB60_EU457075* | 1.99E-04 | 20.025 | 6.143 | 0.004 | 0.010 |
| *Clostridiales_EU453971* | 3.12E-03 | 2.524 | 1.044 | 0.000 | 0.000 |

Statistical analysis was performed using unpaired t tests with Benjamini-Hochberg FDR; differences were considered significant for (q value 0.01).SD (%) : standard deviation.
