# Supplementary material for: Intestinal Sucrase as a Novel Target Contributing to the Regulation of Glycemia by Prebiotics
Source: PLoS One. 2016 Aug 17;11(8):e0160488. doi: 10.1371/journal.pone.0160488 (PMC4988693; doi:10.1371/journal.pone.0160488)
Supplement: S1 Text — (DOCX) [file pone.0160488.s002.docx]

**S1 Text. Materials and Methods**

16S rDNA pyrosequencing

PCR-amplification of the V1-V3 region of the 16S rDNA and library preparation were performed with the following primers (with Illumina overhand adapters), forward (5’-TCGTCGGCAGCGTCAGATGTGTATAAGAGACAG-3’) and reverse (5’-GTVTVGTGGGCTCGGAGATGTGTATAAGAGACAG-3’). Each PCR product was purified with the Agencourt AMPure XP beads kit (Beckman Coulter, Pasadena, USA) and submitted to a second PCR round for indexing, using the Nextera XT index primers 1 and 2. After purification, PCR products were quantified using the Quant-IT PicoGreen (ThermoFisher Scientific, Waltham, USA) and diluted to 10 ngµL-1. A final quantification, by qPCR, of each sample in the library was performed using the KAPA SYBR® FAST qPCR Kit (KapaBiosystems, Wilmington, USA) before normalization, pooling and sequencing on a MiSeq sequencer using v3 reagents (ILLUMINA, USA).

Sequence reads processing were used as previously described [1] using respectively MOTHUR software package v1.35 [2]. Pyronoise algorithm and UCHIME algorithm [3] for alignment and clustering, denoising and chimera detection. 16S Reference alignment and taxonomical assignation were based upon the SILVA database (v1.15) of full-length 16S rDNA sequences.

Subsample datasets were obtained and used to evaluate ecological indicators, richness estimation (Chao1 estimator), microbial biodiversity (reciprocal Simpson index), and the population evenness (derived from simpson index) using MOTHUR [4]. Population structure and community membership were assessed with MOTHUR using distance matrice based on Bray-Curtis dissimilarity index (a measure of community structure which considers shared OTUs and their relative abundances).

Ordination analysis and 3d plots were performed with Vegan, Vegan3d and rgl packages in R [Dixon, Philip]. Non metric dimensional scaling, based upon the Bray-Curtis dissimilarity matrix was applied to visualize the biodiversity between the groups. AMOVA test was performed to assess the diversity clustering of CT and INU groups of Bray-Curtis matrix using MOTHUR [5]. Statistical differences between bacterial biodiversity, richness and evenness were assessed with unpaired t test using PRISM 6 (Graphpad Software), differences were considered significant for a p-value of less than 0.05. All the biosample raw reads have been deposited at the National Center for Biotechnology Information (NCBI) and are available under de Bioproject ID PRJNA315490

Quantitative polymerase chain reaction (qPCR)

q-PCR was performed with a StepOnePlus Real-Time PCR System and software (Applied Biosystems, Den Ijssel, The Netherlands) using Mesa Fast qPCR™ (Eurogentec, Seraing, Belgium) as previously described [6].

For *Blautia* genus quantification, primers and probes were designed specifically for this study and synthesized by Eurogentec (Liège, Belgium). *Blautia* 16S reference sequences were downloaded from SILVA database were aligned with *Blautia* OTU sequences from this study using CLUSTALW (available online at http://www.ebi.ac.uk/Tools/msa/clustalo/). A minimum for three mismatches between target species and neighbors ensured the specificity for primers and probes (Forward, 5'-AGAAATAGGTGCTAATACCGC -3' ; Reverse, 5'-CATCGTATACCACCGGAG-3' ; Probe, FAM-CGCACAGCTTCGCATGAAG-TAMRA). The standard curve is based upon 10 fold dilution of quantified genomic DNA of *B. coccoides*. The DNA quantification is performed with picogreen targeting double-stranded DNA (Promega, Leiden, The Netherlands) and translated into genome equivalent using the known lenght of the genome. Quantitative real-time PCR were performed on LC480 Lightcycler (Roche) in with Lightcycler 480 Probe master mix for a total reaction volume of 25µl. The amplification were carried out in triplicate with 40 cycles of a 95°C denaturation phase followed by a 60°C annealing phase.

1. Rodriguez C, Taminiau B, Brevers B, Avesani V, Van BJ, Leroux A, et al. (2015) Faecal microbiota characterisation of horses using 16 rdna barcoded pyrosequencing, and carriage rate of clostridium difficile at hospital admission. BMC Microbiol 15: 181. doi: 10.1186/s12866-015-0514-5.

2. Schloss PD, Westcott SL, Ryabin T, Hall JR, Hartmann M, Hollister EB, et al. (2009) Introducing mothur: open-source, platform-independent, community-supported software for describing and comparing microbial communities. Appl Environ Microbiol 75: 7537-7541. doi: 10.1128/AEM.01541-09 .

3. Edgar RC, Haas BJ, Clemente JC, Quince C, Knight R (2011) UCHIME improves sensitivity and speed of chimera detection. Bioinformatics 27: 2194-2200. doi: 10.1093/bioinformatics/btr381.

4. Hunter PR, Gaston MA (1988) Numerical index of the discriminatory ability of typing systems: an application of Simpson's index of diversity. J Clin Microbiol 26: 2465-2466.

5. Martin AP (2002) Phylogenetic approaches for describing and comparing the diversity of microbial communities. Appl Environ Microbiol 68: 3673-3682.

6. Bindels LB, Neyrinck AM, Salazar N, Taminiau B, Druart C, Muccioli GG, et al. (2015) Non Digestible Oligosaccharides Modulate the Gut Microbiota to Control the Development of Leukemia and Associated Cachexia in Mice. PLoS One 10: e0131009. doi: 10.1371/journal.pone.0131009.
